# Supplementary material for: Optogenetic Rescue Reveals Spatiotemporal Rules of Germ-Layer Patterning
Source: bioRxiv. 2025 Dec 11:2025.12.08.693069. Preprint. [Version 1] doi: 10.64898/2025.12.08.693069 (PMC12713756; doi:10.64898/2025.12.08.693069)
Supplement: 1 [file NIHPP2025.12.08.693069v1-supplement-1.pdf]

bioRxiv preprint doi: https://doi.org/10.64898/2025.12.08.693069; this version posted December 11, 2025. The copyright holder for this preprint (which was not certified by peer review) is the author/funder, who has granted bioRxiv a license to display the preprint in perpetuity. It is made available under aCC-BY 4.0 International license.

Supplemental Figures:

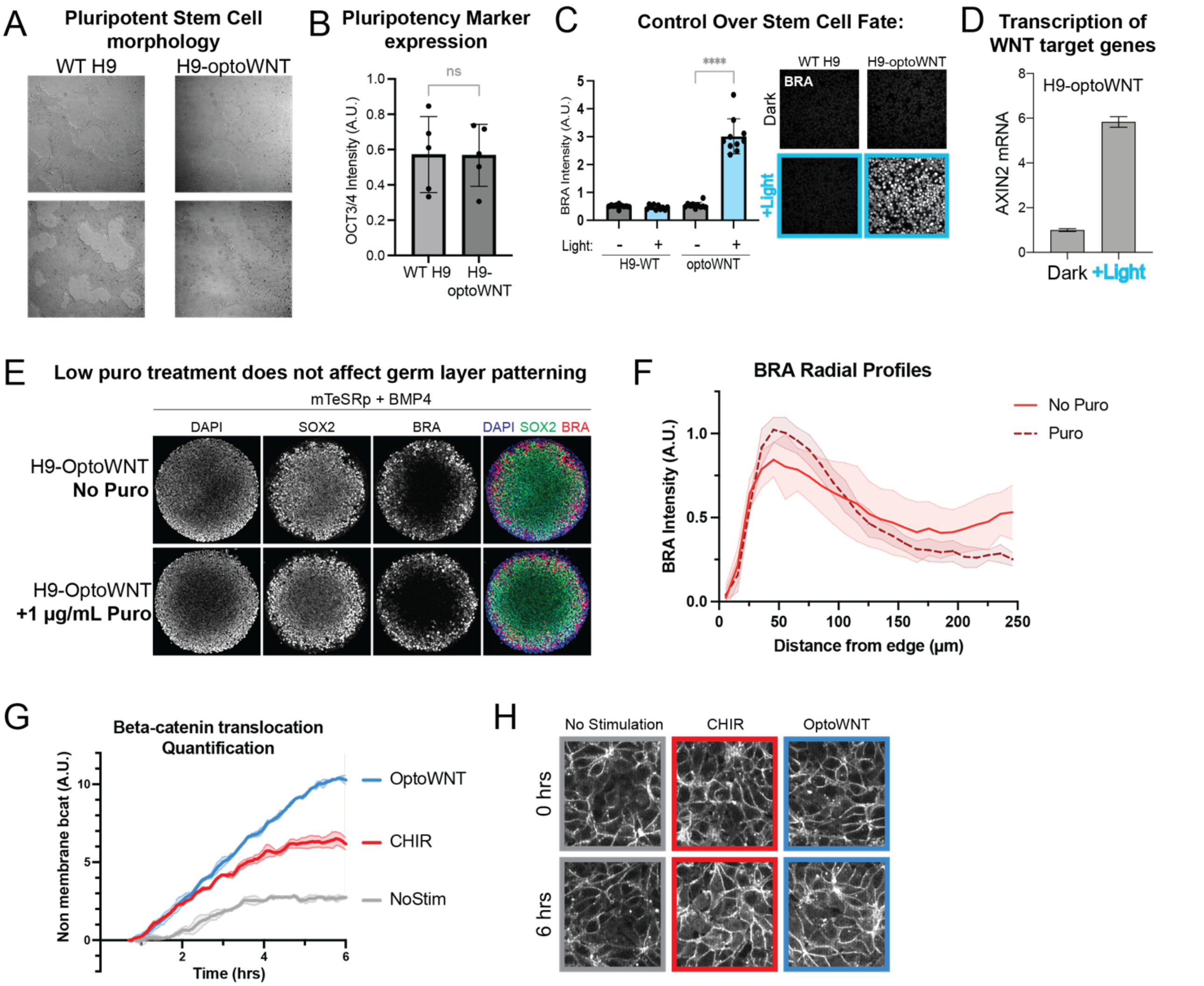

Figure S1. H9-OptoWNT cell line retains pluripotency compared to WT-H9 hESCs and light activation mimics canonical WNT signaling (Related to figure 1) **(A)** Brightfield images of WT H9 cells compared to H9-optoWNT cells. **(B)** OCT3/4 expression detected and quantified via immunofluorescence analysis in WT H9 cells compared to H9-optoWNT cells. **(C)** BRA differentiation in response to blue light stimulation detected via immunofluorescence analysis. **(D)** RT-qPCR of Axin2 mRNA levels in H9-optoWNT cells in response to light stimulation for 24 hours. **(E)** Representative IF stained colonies for H9-optoWNT cells treated with and without puromycin during germ layer formation. **(F)** BRA radial profiles for 5 representative colonies with and without puro treatment, quantified via DAPI nuclear segmentation. (G-H: beta catenin translocation for OptoWNT stimulation compared to CHIR 99021). **(G)** Mean and standard deviation for the centered rolling average of cells treated with 6 hrs of CHIR (red) and OptoWNT (blue) compared to no stimulation (grey). **(H)** Representative fields of view of beta catenin-tdmruby before (top) and after (bottom) 6 hrs of stimulation, no stimulation (*left*), stimulation with CHIR (*middle*), and OptoWNT (*right*).

bioRxiv preprint doi: https://doi.org/10.64898/2025.12.08.693069; this version posted December 11, 2025. The copyright holder for this preprint (which was not certified by peer review) is the author/funder, who has granted bioRxiv a license to display the preprint in perpetuity. It is made available under aCC-BY 4.0 International license.

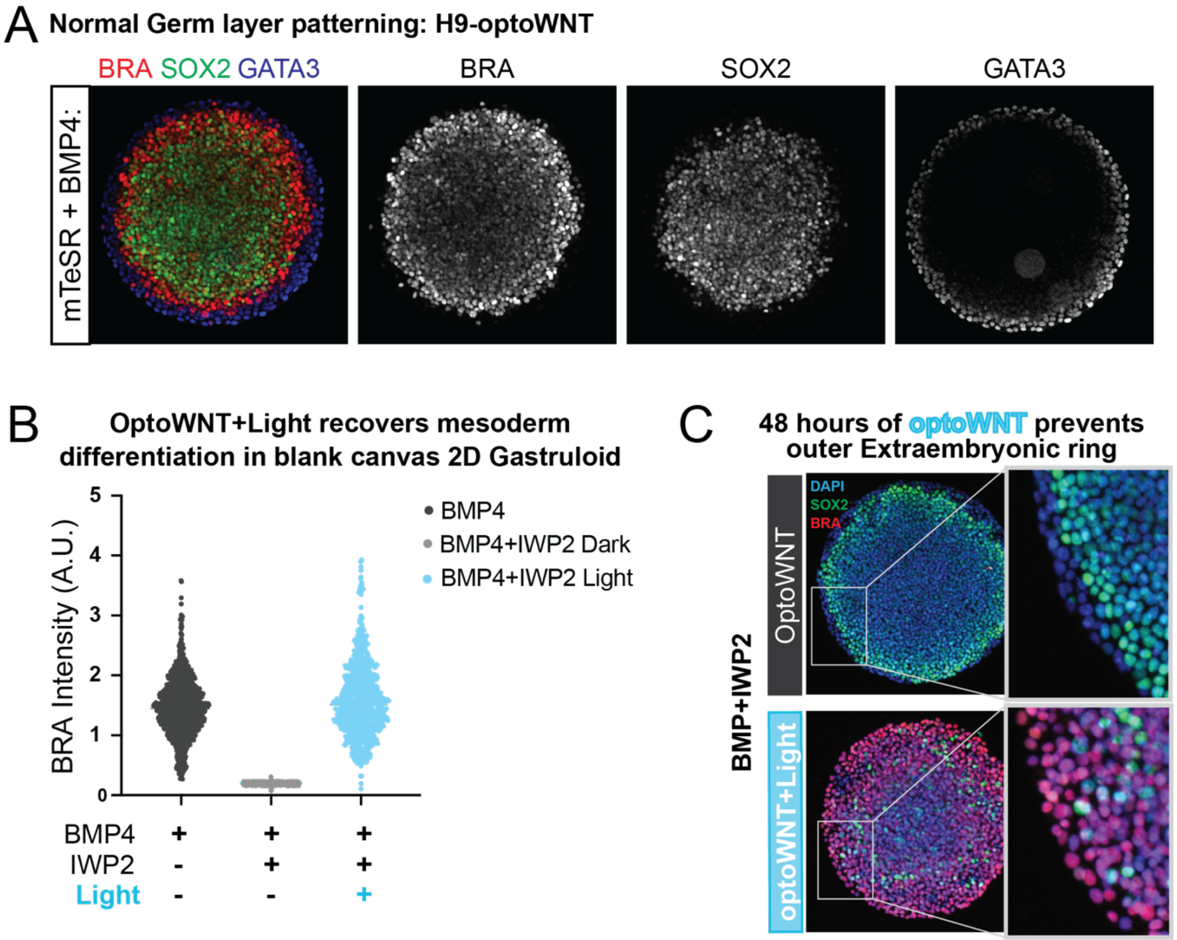

Figure S2. Optogenetic WNT activation recovers mesoderm differentiation but prevents in "blank canvas" 2D gastruloid (Related to figure 1) (A) Individual channels for micropatterned H9-optoWNT treated with BMP4 for 48 hours and stained for the germ layers (colony shown in fig 1C) **(B)** Quantification of Brachyury in blank canvas experiment compared to BMP4 treated control colonies for 3 replicate colonies. BRA intensity quantified via DAPI nuclear segmentation. **(C)** H9-optoWNT colonies treated with BMP and IWP, IF stained for DAPI and indicated fate markers. Boxes highlighting that OptoWNT stimulation for 48 hours prevents differentiation of the outer extraembryonic ring as indicated here by DAPI, SOX2-. (colonies shown in Fig 1I)

bioRxiv preprint doi: https://doi.org/10.64898/2025.12.08.693069; this version posted December 11, 2025. The copyright holder for this preprint (which was not certified by peer review) is the author/funder, who has granted bioRxiv a license to display the preprint in perpetuity. It is made available under aCC-BY 4.0 International license.

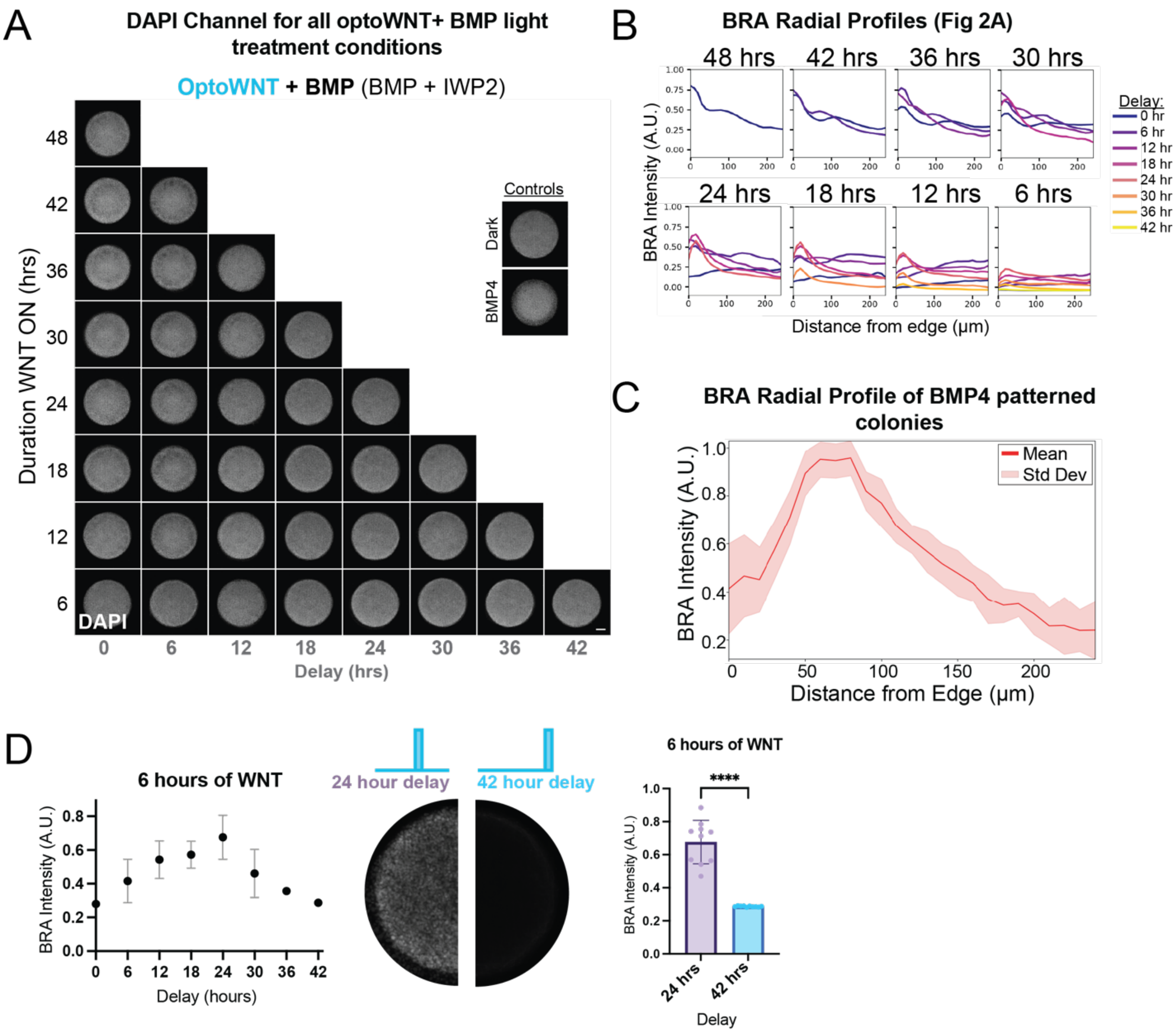

Figure S3. The timing of OptoWNT modulates mesoderm differentiation in Gastruloids treated with BMP+IWP2 (Related to Figure 2) **(A)** Colony averages of DAPI channel for all colonies shown in Fig 2A. 10 replicate colonies per treatment. **(B)** BRA radial profiles for each light treatment in Fig 2A. **(C)** BRA radial profile of BMP4 patterned colonies (10 replicate colonies). **(D)** Representative light treatments demonstrating that the timing of the WNT signal affects the amount of mesoderm differentiation for 6 hours of OptoWNT. *Left*: BRA differentiation for 6 hours of WNT at each delay indicated. *Middle*: BRA colony averages with 24- and 42-hour delays. *Right*: differences in BRA differentiation at 24 and 42 hour delays is statistically significant ****P<0.0001

bioRxiv preprint doi: https://doi.org/10.64898/2025.12.08.693069; this version posted December 11, 2025. The copyright holder for this preprint (which was not certified by peer review) is the author/funder, who has granted bioRxiv a license to display the preprint in perpetuity. It is made available under aCC-BY 4.0 International license.

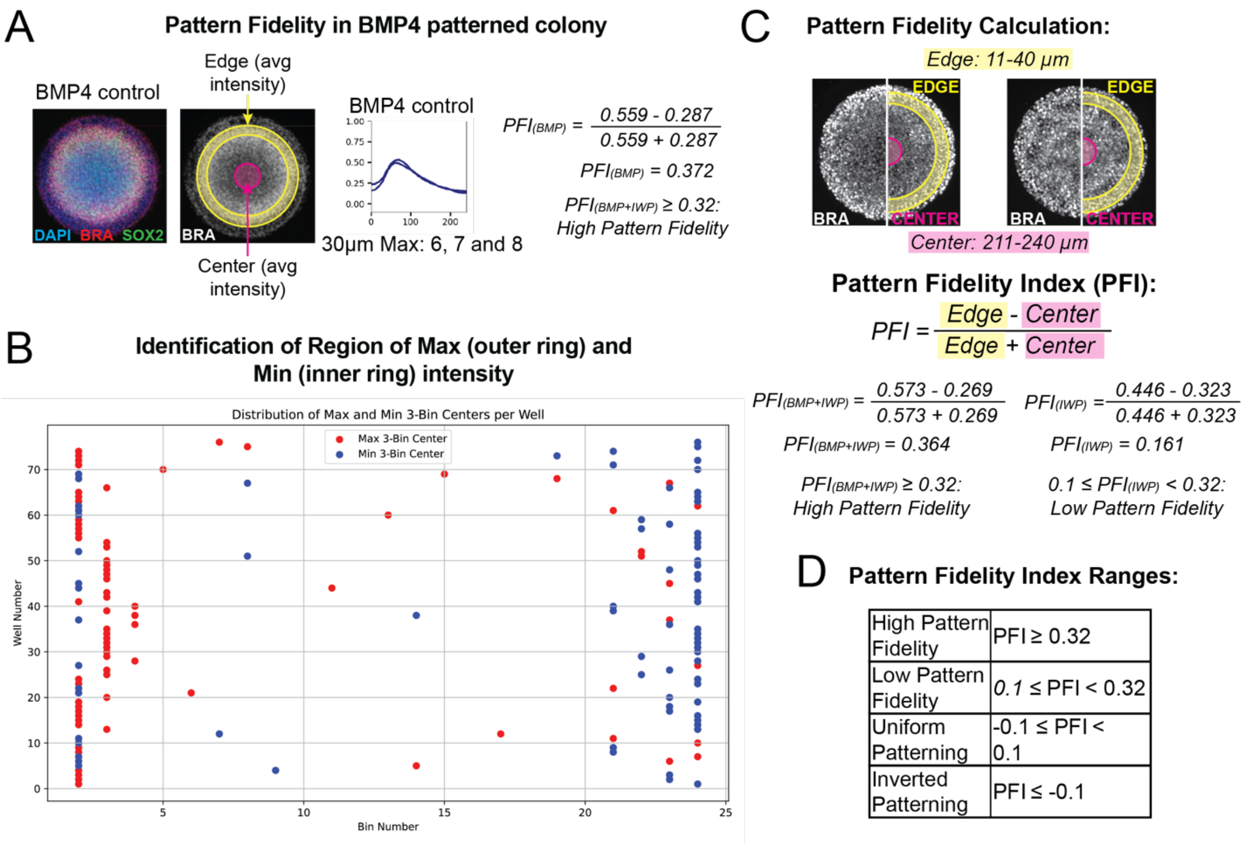

Figure S4. The Pattern Fidelity index is a metric that reflects the expected difference in mesoderm differentiation at the edge compared to the center of the colony. (A) In a BMP4 patterned 2D gastruloid, Brachyury patterns in a ring that is a few cell widths in from the edge of the colony. The region of max BRA intensity is in this ring (yellow), and the BRA signal in the center of the colony should be much lower (magenta). Calculation (*left*): PFI for BMP4 patterned colonies results in high pattern fidelity. **(B)** Visualization of the 3-bin max (red dots) and 3-bin min (blue dots) for each light treatment to determine the “edge” and “center” regions for optogenetically patterned colonies. **(C)** Pattern fidelity calculations for the optoWNT patterned colonies shown in Fig 3C. These gastruloids treated with OptoWNT+BMP (*left*) result in “high” pattern fidelity, while those treated with OptoWNT alone (*right*) result in low pattern fidelity.

bioRxiv preprint doi: https://doi.org/10.64898/2025.12.08.693069; this version posted December 11, 2025. The copyright holder for this preprint (which was not certified by peer review) is the author/funder, who has granted bioRxiv a license to display the preprint in perpetuity. It is made available under aCC-BY 4.0 International license.

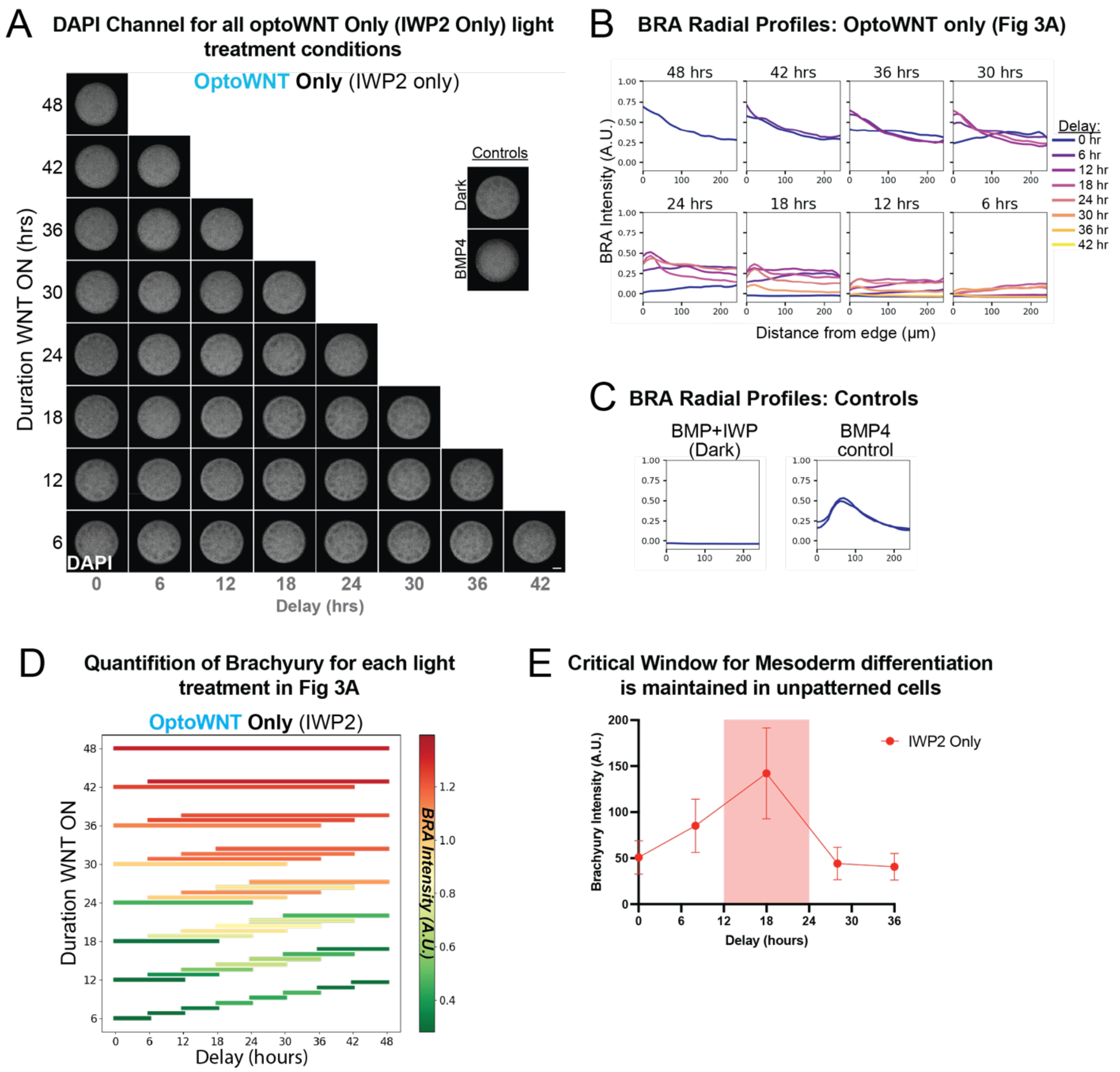

Figure S5. The critical window for mesoderm differentiation exists without BMP4 and in monolayer culture. (Related to Figure 3) **(A)** Colony averages of DAPI channel for all colonies shown in Fig 3A. 10 replicate colonies per treatment. **(B)** BRA radial profiles for each light treatment in Fig 3A. **(C)** BRA radial profile of control patterned colonies (10 replicate colonies per line). **(D)** Heat map bar plot representing the average mesoderm differentiation for each light treatment over 10 replicate colonies shown in 3A. The color of each bar represents the intensity of Brachyury (red is the highest intensity and green is the lowest). The length and location of each bar represents the duration and timing of the WNT signal respectively. The mean level of mesoderm differentiation per light treatment was quantified based on DAPI nuclear segmentation. **(E)** Quantification of Brachyury for H9- optoWNT cells plated in monolayer (not micropatterned) culture, and treated with 12 hrs of OptoWNT at the indicated delays. Detected via IF and DAPI nuclear segmentation.

bioRxiv preprint doi: https://doi.org/10.64898/2025.12.08.693069; this version posted December 11, 2025. The copyright holder for this preprint (which was not certified by peer review) is the author/funder, who has granted bioRxiv a license to display the preprint in perpetuity. It is made available under aCC-BY 4.0 International license.

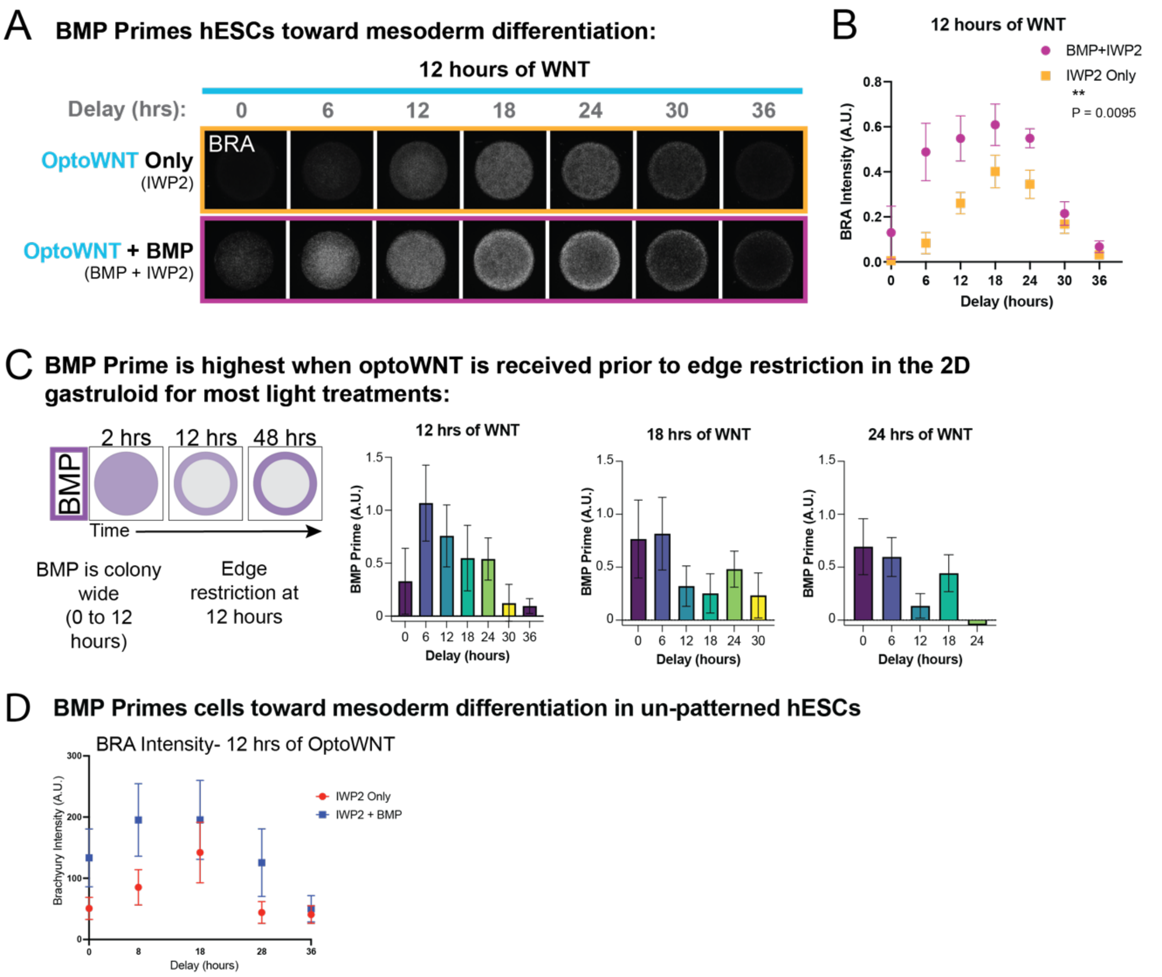

Figure S6. BMP primes cells toward mesoderm differentiation through some other mechanism besides WNT ligand secretion (Related to Figure 3) **(A)** Comparison of gastruloids treated with 12 hours of blank canvas OptoWNT at different delays, with and without BMP, demonstrating the increase in mesoderm differentiation when BMP4 is present despite receiving the same duration of WNT and WNT ligand secretion inhibited. Colony average of 10 replicate colonies. **(B)** Quantification of Brachyury for the colonies shown in (A). (C) *Left*: schematic demonstrating the timing of BMP signaling edge restriction that occurs at around 12 hours. The BMP prime is generally more pronounce when the WNT signal is received prior to edge restriction. **(D)** BMP primes cells toward WNT driven mesoderm differentiation in unpatterned cells. H9- optoWNT cells plated in monolayer (not micropatterned) culture, and stimulated with 12 hrs of OptoWNT at the indicated delays, treated with optoWNT Only (IWP2 only, red) and OptoWNT + BMP (IWP2+BMP, blue).

bioRxiv preprint doi: https://doi.org/10.64898/2025.12.08.693069; this version posted December 11, 2025. The copyright holder for this preprint (which was not certified by peer review) is the author/funder, who has granted bioRxiv a license to display the preprint in perpetuity. It is made available under aCC-BY 4.0 International license.

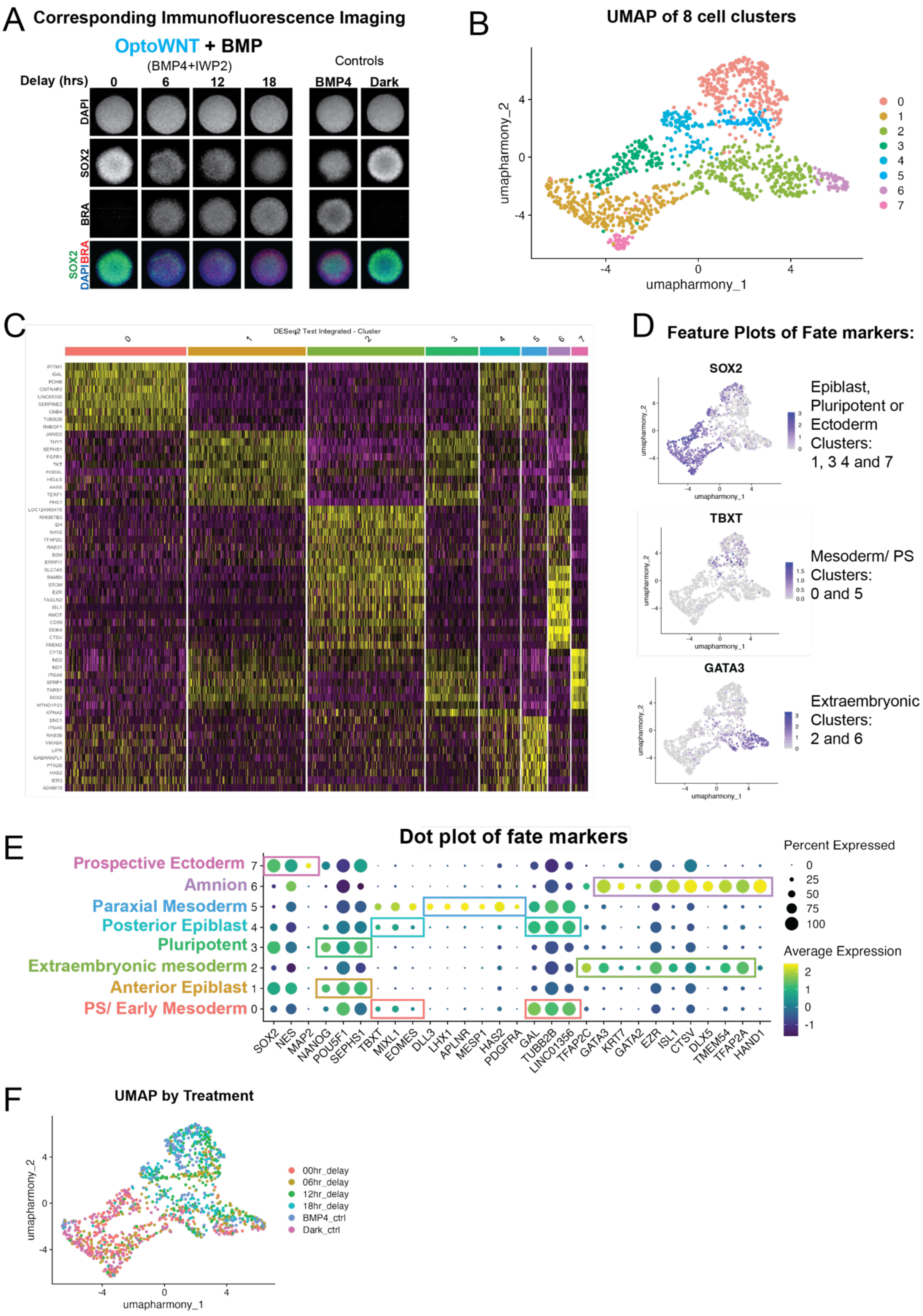

bioRxiv preprint doi: https://doi.org/10.64898/2025.12.08.693069; this version posted December 11, 2025. The copyright holder for this preprint (which was not certified by peer review) is the author/funder, who has granted bioRxiv a license to display the preprint in perpetuity. It is made available under aCC-BY 4.0 International license.

Figure S7. Corresponding Immunofluorescence analysis and scRNAseq cluster cell type annotation (Related to figure 4) **(A)** Corresponding immunofluorescence analysis for each treatment included in the scRNAseq experiment. Colony averages of 10 replicate colonies. The same LUTS were used for all conditions. **(B)** The 8 cell clusters identified via unsupervised clustering and corresponding cluster numbers. **(C)** Differential gene expression across the 8 cell clusters. **(D)** Feature plots of fate markers identified clusters as extraembryonic (clusters 2 and 6), mesoderm or primitive streak lineage (clusters 0 and 5), or epiblast/ pluripotent/ ectoderm (clusters 1, 3, 4, and 7). **(E)** Dot plot of key marker genes to further elucidate the identity of each cluster. **(F)** UMAP colored by treatment.

bioRxiv preprint doi: https://doi.org/10.64898/2025.12.08.693069; this version posted December 11, 2025. The copyright holder for this preprint (which was not certified by peer review) is the author/funder, who has granted bioRxiv a license to display the preprint in perpetuity. It is made available under aCC-BY 4.0 International license.

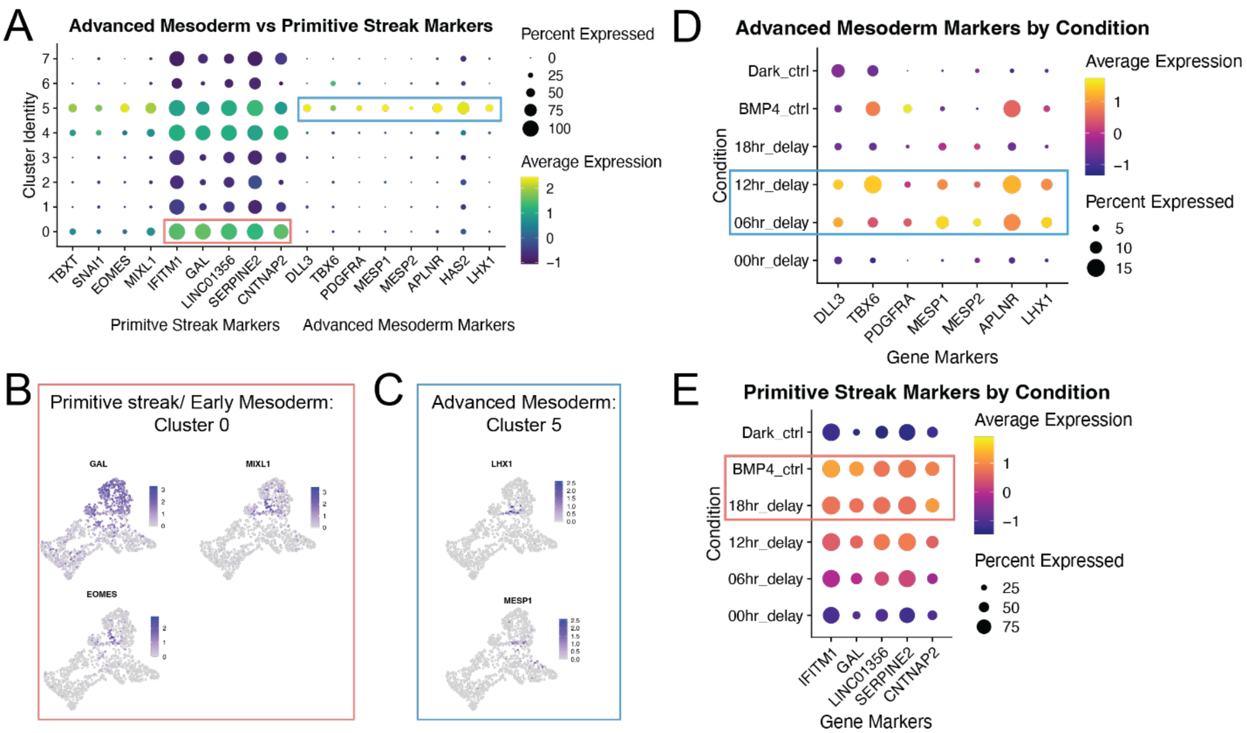

Figure S8. OptoWNT patterned 2D gastruloids with 6- and 12- hour delays differentiate cells into advanced mesoderm subtypes (Related to figure 4) **(A)** Dot plot of advanced mesoderm and primitive streak markers distinguishing cluster 5 as a more advanced paraxial mesoderm population and cluster 0 as an early mesoderm/ primitive streak population. **(B)** Feature plots of primitive streak and early mesoderm marker genes. **(C)** Feature plots of more advanced paraxial and intermediate mesoderm markers. **(D)** Dot plot of advanced mesoderm markers by condition. The 12- and 6- hour delays (aberrant patterning phenotypes) result in high expression of advanced (paraxial, lateral plate and intermediate) mesoderm subtypes (percent is of all cells from that treatment, not cluster). **(E)** Dot plot of primitive streak/ early mesoderm markers by condition. BMP4 (control) and partial rescue (12 hrs optoWNT with 18-hour delay) patterned colonies express higher levels of primitive streak marker genes. These genes are expressed at much lower levels in the aberrant patterning phenotypes (0-, 6-, and 12-hour delays).

bioRxiv preprint doi: https://doi.org/10.64898/2025.12.08.693069; this version posted December 11, 2025. The copyright holder for this preprint (which was not certified by peer review) is the author/funder, who has granted bioRxiv a license to display the preprint in perpetuity. It is made available under aCC-BY 4.0 International license.

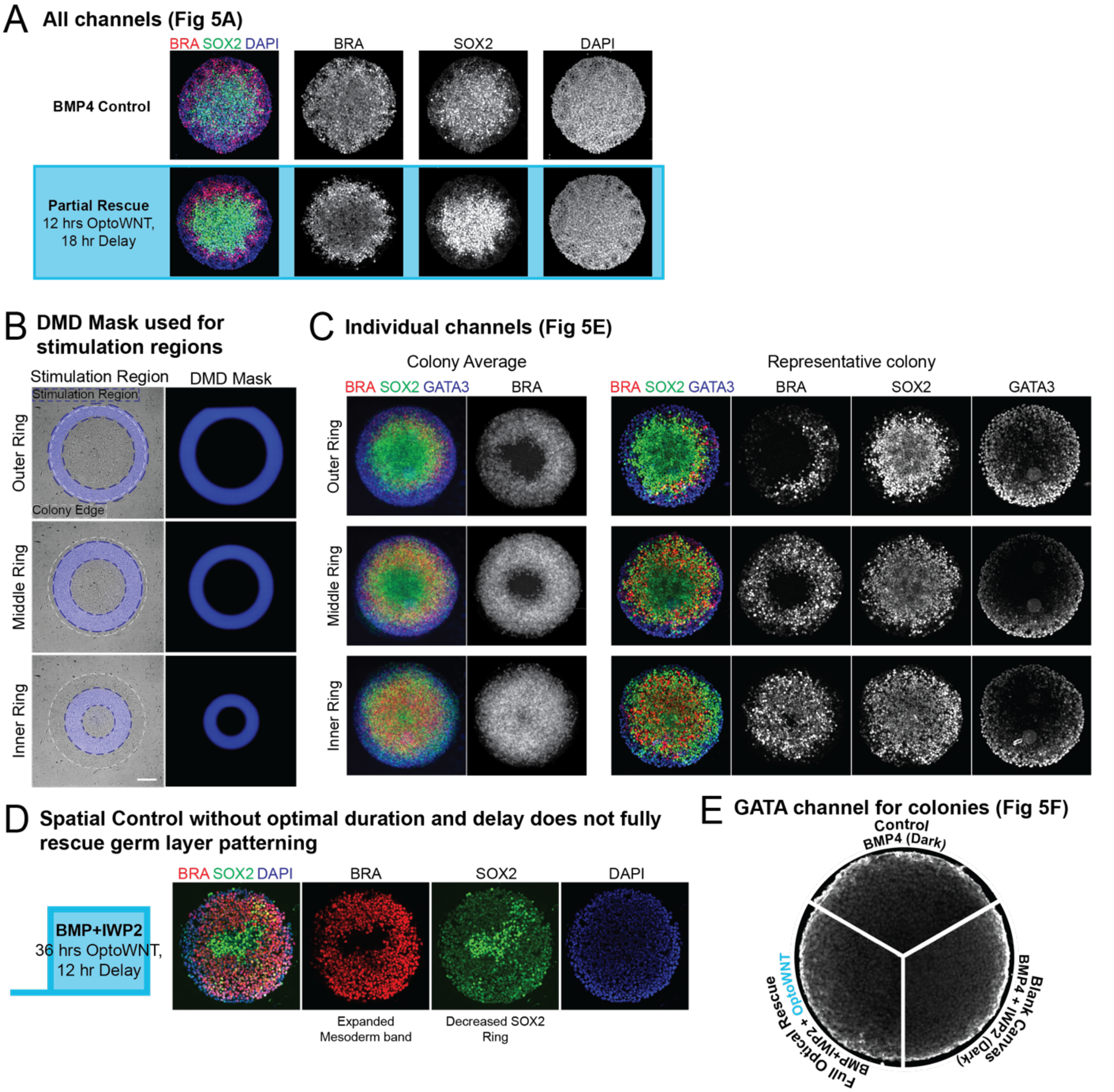

Figure S9. Spatiotemporal control of OptoWNT rescues BRA and GATA3 patterning (Related to figure 5) **(A)** All channels shown in antibody-stained colony in figure 5A highlighting the differences in 2D gastruloid boundary formation in a normally patterned BMP4 colony compared to the partial optical rescue. **(B)** The DMD masks used for each stimulation region. **(C)** Additional channels for the colony average and representative colonies shown in 5E. **(D)** Gastruloids patterned with spatial control (middle ring stimulation region) but stimulated with a non-optimal duration and delay results a mesoderm band that is too wide and a decreased SOX2+ inner ring. **(E)** GATA3 channel for the colonies shown in figure 5F.

bioRxiv preprint doi: https://doi.org/10.64898/2025.12.08.693069; this version posted December 11, 2025. The copyright holder for this preprint (which was not certified by peer review) is the author/funder, who has granted bioRxiv a license to display the preprint in perpetuity. It is made available under aCC-BY 4.0 International license.

Supplementary Notes:

Supplementary Note 1: Annotation of cell types in scRNAseq clusters

Unannotated clusters (also shown in **Fig S7B**):

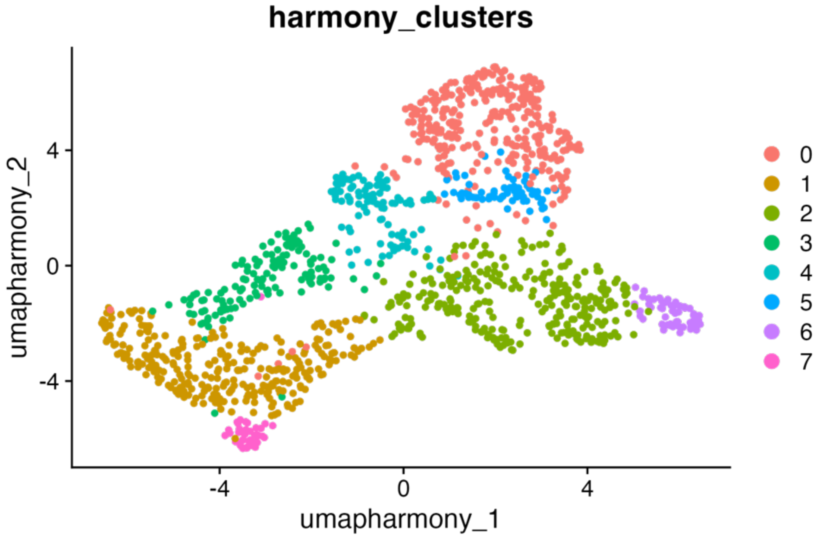

There were 2 clusters, cluster 0 and cluster 5 (**Fig S7B**), that clearly corresponded to mesodermal lineages as indicated by expression of TBXT (**Fig S7E**). The cluster that we identified as primitive streak/ early mesoderm (cluster 0) is enriched for canonical primitive streak markers (TBXT, MIXL1, EOMES) and genes that are highly expressed in primitive streak and midline mesoderm in a CS7 embryo (GAL, SERPINE2, CNTNAP2)[38] (**Fig S7E**). There is some expression of SOX2 in this cluster while the other mesodermal population (cluster 5) has negligible SOX2 expression (**Fig S7D**) supporting a primitive streak identity rather than a committed advanced mesoderm population. Therefore, we identify this population as primitive streak and possibly primed for midline mesoderm fates such as the notochord and axial mesoderm. Co-expression of both primitive streak and mesoderm markers led to the identification of PS/early mesoderm representing the forming mesoderm.

The cluster identified as Paraxial and lateral plate mesoderm (cluster 5) is strongly enriched for a mesodermal population that has already traversed the primitive streak (LIFR, ENC1, APLNR, LHX1, MESP1)[12] and is highly migratory (RAB3B, PTK2B, ITGA5) (**Supp Table 3**). DLL3, TBX6, MESP1 and MESP2 are paraxial mesoderm markers[47] that are all expressed in this cluster. There is also some expression of lateral plate markers including APLNR[64], suggesting this cluster could contain subclusters of both paraxial and lateral plate mesoderm. However, the paraxial signature is stronger (Fig S8D). This cluster also has much lower expression of pluripotency markers (SOX2, POU5F1 and SEPHS1) (**Fig S7E**) and highly expresses genes involved in ECM remodeling and integrin signaling which are hallmarks of EMT[65] further supporting the paraxial/ lateral plate mesoderm identity.

The genes expressed in two of the clusters were consistent with extraembryonic identities (GATA3, ISL1, and EZR). In one of the clusters, HAND1 is specifically and significantly upregulated supporting an amnion identity[66]. Compared to the amnion cluster, expression of extraembryonic specific markers is lower in the other cluster, and the top differentially expressed genes suggest a high BMP signature (BMP4, BAMB, ID4), and reveal some ECM and motility genes characteristic of extraembryonic mesoderm (HPGD, SEM6D)[67,68]. Extraembryonic mesoderm is in contact with amnion in a gastrulating human embryo, and was recently identified

bioRxiv preprint doi: https://doi.org/10.64898/2025.12.08.693069; this version posted December 11, 2025. The copyright holder for this preprint (which was not certified by peer review) is the author/funder, who has granted bioRxiv a license to display the preprint in perpetuity. It is made available under aCC-BY 4.0 International license.

in 2D gastruloids[39,40,69], leading to the identification of the other extraembryonic lineage as extraembryonic mesoderm.

We found three clusters that highly express SOX2 (Clusters 7, 3 and 1: **Fig S7E**). The gene expression patterns of these three clusters suggest they all retain pluripotency rather than representing specified ectoderm. Cluster 7 highly expresses MAP2[70], SFRP1[71] and NES[72] and has lower pluripotency marker expression (POU5F1, NANOG, SEPHS1) than clusters 3 and 1, suggesting neuro-ectoderm. However, the pluripotent and metabolically active signature of the top differentially expressed genes (**Supp table 3**) suggests this population is not yet committed to ectodermal fates leading to the *prospective* neuro-ectoderm identification.

Cluster 3 highly expresses SOX2 and other pluripotency markers but does not significantly upregulate any posterior or anterior epiblast identity genes and exhibits lower expression of neuroectoderm marker NES than cluster 1. The top differentially expressed genes in this cluster (**Supp table 3**) contain pluripotent and proliferative genes suggesting a pluripotent epiblast population not primed for either anterior or posterior fates. Additionally, cells in this cluster are only present in the dark (no WNT) and 0 hr delay (no mesoderm) treatment conditions (**Fig 4E**), and are not present in BMP4 patterned colonies, further supporting the pluripotent identity of this cluster.

Cluster 1 highly expresses pluripotency and epiblast markers (SOX2, NANOG, POU5F1, and SEPHS1), in addition cell cycle genes (**Supp table 3**). It also expresses neuroectoderm markers such as NES suggesting this population has not been exposed to posteriorizing cues, and leading to the identification of epiblast primed for anterior fates identity. SOX2 expression is also upregulated in Cluster 4 (**Fig S7E**), and this cluster expresses primitive streak markers TBXT, MIXL1, EOMES, GAL, and TUBB2B at lower levels than the mesodermal populations. The top differentially expressed genes (**Supp table 3**) are suggestive of a highly proliferative and more pluripotent population than clusters 0 and 5, leading to the identification of epiblast primed to ingress through the primitive streak, or “posterior primed epiblast”.
